# Progress on the Prevention and Treatment of Hantavirus Disease

**DOI:** 10.3390/v11070610

**Published:** 2019-07-04

**Authors:** Rebecca L. Brocato, Jay W. Hooper

**Affiliations:** Virology Division, United States Army Medical Research Institute of Infectious Diseases, Frederick, MD 21702, USA

**Keywords:** hantavirus, hemorrhagic fever with renal syndrome, hantavirus pulmonary syndrome, vaccine, antiviral

## Abstract

Hantaviruses, members of the order *Bunyavirales*, family *Hantaviridae*, have a world-wide distribution and are responsible for greater than 150,000 cases of disease per year. The spectrum of disease associated with hantavirus infection include hemorrhagic fever with renal syndrome (HFRS) and hantavirus pulmonary syndrome (HPS) also known as hantavirus cardiopulmonary syndrome (HCPS). There are currently no FDA-approved vaccines or treatments for these hantavirus diseases. This review provides a summary of the status of vaccine and antiviral treatment efforts including those tested in animal models or human clinical trials.

## 1. Introduction

Hantaviruses are the etiological agents responsible for two disease syndromes: Hemorrhagic fever with renal syndrome (HFRS) and hantavirus pulmonary syndrome (HPS) or hantavirus cardiopulmonary syndrome (HCPS) [1]. Old World hantaviruses, including the prototypic hantavirus Hantaan virus (HTNV), Puumala virus (PUUV), and Dobrava virus (DOBV), found predominantly in Europe and Asia, cause HFRS with a case fatality rate ranging from <1 to 15% depending on the infecting virus [2]. Seoul virus (SEOV), an HFRS-causing hantavirus carried by the Norway rat (*Rattus norvegicus*) has a widespread distribution coinciding with transportation of this rodent reservoir worldwide [3]. New World hantaviruses include Andes virus (ANDV) and Sin Nombre virus (SNV), found predominantly in the Americas, and Choclo virus (CHOV) found in Central America, causing HPS with a case fatality rate of up to 40% [4]. Human infections from these pathogenic hantaviruses are typically restricted to the distribution of the rodent reservoir. To date, ANDV is the only known hantavirus with demonstrated person-to-person transmission [5].

Hantaviruses are enveloped, negative-sense, single-stranded RNA viruses with tripartite genome. The three segments are denoted as small (S), medium (M), and large (L) encoding for the nucleoprotein (N), glycoproteins (G_n_ and G_c_), and the RNA-dependent RNA polymerase, respectively [1]. Hantaviruses infect microvascular endothelial cells altering the barrier properties of these cells resulting in a vascular leakage disease in the target organ, the kidney for HFRS- and lung for HPS-causing hantaviruses [6]. It is important to note that there are many shared clinical signs between infection with HFRS- and HPS-causing hantaviruses as well as overlap in the target organ (i.e., kidney failure in severe cases of HPS and lung disease in cases of HFRS) [7,8,9].

Reported cases of HFRS exceed 150,000 per year [10]. The majority of HFRS cases are reported in Asia, specifically China, resulting from infection with HTNV (carried by *Apodemus agrarius*) and SEOV with greater than 1.5 million cases between 1950 and 2007 [11]. Cases of HTNV are also reported in Russia and Korea. DOBV (carried by *Apodemus flavicollis*) is associated with severe HFRS disease in Europe while PUUV (carried by *Myodes glareolus*) is associated with a less severe disease in Europe, particularly Scandinavia and western Russia [10]. In comparison to HFRS, far fewer cases of HPS have been reported, with 624 cases over a 20-year period in the United States [12] and 109 cases reported in Canada from 1994 to 2014 [13], the vast majority of these caused by SNV (carried by *Peromyscus maniculatus*) infection. ANDV (carried by *Oligoryzomys longicaudatus*) is endemic in South America with 786 cases reported in Chile between 1995 and 2012 [14] and 1243 cases reported in Argentina between 1995 and 2017 [15,16]. In Brazil, 884 cases of HPS were reported from 1993 to 2007 [17], with approximately 1600 cases reported through 2013 [18]. Of these documented cases, most are associated with Araraquara virus infection (carried by *Oligoryzomys nigripes*). Sporadic cases of HPS have also been reported in Uruguay, Paraguay, Bolivia, Peru, Venezuela, and Panama [10]. Humans become infected with these viruses through accidental inhalation or ingestion of excreta or secreta, or bite. Given the worldwide distribution of hantaviruses, there is a need for medical countermeasures for the prevention and treatment of hantavirus diseases. There are no current FDA-approved vaccines or antivirals for hantavirus infection. This review serves as a summary of the current status of hantavirus vaccine and antiviral development based on animal model testing and human clinical trials.

## 2. Hantavirus Disease Vaccine Development

### 2.1. Approved HFRS Vaccines in China and Korea

HFRS cases in China have been reported in 29 of 31 provinces sparking the implementation of widespread preventative measures to include a vaccination strategy. Inactivated vaccines developed in cell culture have evolved to the bivalent vaccine for HTNV and SEOV made in Vero cells currently in use [11]. Approximately 2 million vaccine doses are administered annually and is offered as part of the national Expanded Program on Immunization. Following the initial vaccination scheme of 3 doses at 0 days, 15 days, and 1 year, 90% of subjects retained positive rates of neutralizing antibody titers 33 months after the boost [19]. Cases of HFRS in the Republic of Korea also resulted in development of a vaccine. Hantavax^®^ is a formalin-inactivated vaccine prepared by HTNV growth in suckling mouse brain. While eliciting strong responses immediately after the boost (97% seroconversion 30 days following the boost), only 50% of volunteers had neutralizing antibodies 1 year following the boost [20]. Increased responses have been noted in a 3-dose strategy [21,22]; however, it has been noted that Hantavax^®^ vaccination fails to demonstrate a statistically significant reduction in HFRS disease severity [23]. Vaccination, in addition to other preventative measures, have decreased the number of cases of HFRS in these regions [24].

### 2.2. Virus-Like Particles (VLP) Vaccines

Previous research has demonstrated that S and M or only M expressed gene products assemble into virus-like particles (VLP) that resemble hantavirus virions when examined by electron microscopy [25,26]. In China, in an effort to abrogate safety concerns with the inactivated whole virus vaccine, sequences of the membrane-bound forms of immune-stimulating molecules CD40 ligand (CD40L) and granulocyte macrophage colony-stimulating factor (GM-CSF) were incorporated into vectors along with HTNV S and M segments and VLPs were generated in vitro [27]. Enhanced HTNV-specific antibody and neutralizing antibody were observed in VLP-immunized mice, in addition to significantly reducing viral load in organs after challenge. The long-term protective efficacy of the HTNV CD40 GM-CSF VLPs were addressed after 2 doses and HTNV challenge 6 months following showed high neutralizing antibody titers in mice [28].

Another VLP approach involves the insertion of foreign protein segments into the core protein of hepatitis B virus (HBcAg) to induce humoral and cellular responses [29]. For hantavirus vaccine development, a segment of the PUUV Vranica/Hällnäs strain N protein (aa 1-45) administered with and without adjuvant was efficacious to prevent PUUV infection in bank voles [30,31,32]. Interestingly, PUUV strain CG18–20 N protein (aa 1–45) inserted into the c/e1 internal region (at aa 78) of HBcAg was highly immunogenic and protective in bank voles [32]. Taken together, these studies have optimized the insertion site in HBcAg and identified two protective regions in the N protein. Based on these initial findings, similarly generated DOBV and HTNV chimeric VLPs were generated and shown to be immunogenic when administered without adjuvant, all IgG subclasses were induced, and cross-reactive against multiple strains of DOBV, HTNV, and PUUV in two mouse models [33,34]. Unfortunately, vaccine protective efficacy was not evaluated in these two studies.

### 2.3. Recombinant Proteins

Given the immunogenicity of the N, G_n_, and G_c_ protein, recombinant proteins were generated and tested as potential vaccine candidates. Using a baculovirus expression system, recombinant N, G_n_, and G_c_ proteins were used to immunize hamsters prior to HTNV infection [35]. Partial protection from infection was observed when hamsters were immunized with recombinant G_n_, and G_c_ proteins independently and full protection was observed when hamsters were immunized with a combination G_n_/G_c_ or N proteins. Also using a baculovirus expression system, truncated recombinant N proteins were developed indicating the amino terminal region is required for protection, harboring six of seven epitopes analyzed using bank vole monoclonal antibodies [36]. Similarly, recombinant N proteins for hantaviruses have been generated in *Saccharomyces cervisiae* that were protective in the PUUV/bank vole model [37]. Cross-reactivity was evaluated with PUUV, ANDV, and DOBV recombinant N proteins generated in *Escherichia coli* cells. PUUV-infected bank voles were completely protected when immunized with PUUV N, partially protected when immunized with ANDV N, and not protected when immunized with DOBV N [38]. PUUV and ANDV N proteins share 73% identity at the amino acid level, indicating several shared antigenic regions.

German researchers used a similar approach to develop a His-tagged DOBV recombinant N proteins in *Saccharomyces cervisiae*. Immunogenicity, but not protective efficacy, was evaluated in two mouse models showing N-specific antibody titers 8 months following a 3-dose vaccination strategy, N-specific antibody induction across all IgG subclasses, and strong cross-reactivity against HTNV [39]. While in Belgium, truncated and full-length His-tagged DOBV recombinant N proteins expressed in *Escherichia coli* were coupled to the outer membrane protein A derived from *Klebsiella pneumoniae* (rP40) protected mice immunized 3 times from a DOBV challenge [40]. The same strategy was used to develop a full-length and truncated PUUV recombinant N using the same rP40 carrier molecule; however, only the truncated construct linked to rP40 was 100% protective in mice challenged with PUUV [41].

### 2.4. Virus-Vectored Recombinant Vaccines

A double recombinant, chimeric molecular vaccine was developed by inserting HTNV S and M segment cDNA into vaccinia virus (VACV) [42]. Protective efficacy of this vaccine was initially evaluated in Syrian hamsters. Two doses of the VACV-vectored HTNV vaccine protected all hamsters infected with either HTNV or SEOV, but not PUUV [43]. This vaccine was further evaluated in both a Phase 1 and Phase 2 clinical trial [44]. Results of the Phase 1 trial indicate that the vaccine was deemed safe, neutralizing antibody titers to both VACV and HTNV increased following a second vaccination, and subcutaneous inoculation was superior to scarification as a vaccine delivery route. Based on the Phase 1, a Phase 2 was conducted demonstrating HTNV neutralizing antibodies were detected in 72% of VACV-naïve volunteers in comparison to 26% in VACV-immune volunteers. Continued efforts to advance this vaccine were halted based on pre-existing VACV immunity.

Nonreplicating adenovirus vectors expressing ANDV N, G_n_, G_c_, or G_n_ + G_c_ were able to elicit robust CTL responses in mice and were protective when hamsters were challenged with a uniformly lethal dose of ANDV [45]. While promising in animal models, human pre-existing immunity to adenovirus type 5 remains a substantial hurdle for this type of vaccine.

Vesicular stomatitis virus (VSV) pseudotype virus containing hantavirus glycoproteins was examined as a potential vaccine strategy. A nonreplicating VSV pseudotype containing HTNV G_n_ and G_c_ was immunogenic and protective against a HTNV challenge in mice after 3 doses [46]. Similarly, VSV containing the ANDV glycoprotein precursor (GPC) effectively prevented lethality in the ANDV/hamster model after a single dose [47]. Interestingly, in this study, the timing of vaccine administration played a role in how the vaccine protects. Four weeks after vaccination, hamsters developed a robust neutralizing antibody response and protection from ANDV challenge suggestive of sterile immunity. By reducing the timing between vaccination and challenge to 1 week, protection is attributed to innate immune responses. The long-term efficacy of this vaccine was evaluated. Vaccinated hamsters were significantly protected from ANDV lethality 6 months, but not 1 year following a single dose of VSVΔG-ANDV-GPC [48].

### 2.5. Nucleic Acid-Based Molecular Vaccines

In an effort to prime a CD4+ T cell response, a Chinese group developed a DNA vaccine encoding the HTNV G_n_ and lysosomal-associated membrane protein 1 (LAMP1) [49]. LAMP1 alters the antigen processing pathway to major histocompatibility complex II, resulting in enhanced humoral immune responses. Immune responses to 3 doses of vaccine were maintained over 6 months in mice and mice were protected from HTNV challenge 4 months following the first vaccination [50]. Similar results were obtained with a vaccine utilizing G_c_ rather than G_n_ [51]. The overarching goal of these studies is to improve upon the immunological memory that is lacking in the inactivated vaccine currently used in China. These initial studies indicate that the DNA vaccine, pVAX-LAMP/G_n_ has the potential to achieve this goal.

DNA vaccines targeting the S and M segments of SEOV were generated by either cloning the gene segment into the DNA plasmid expression vector pWRG7077 or into a DNA-based Sindbis replicon or packaged Sindbis replicon vector [52,53]. Increased protection from SEOV infection was observed with M segment vaccination in Syrian hamsters. It should also be noted that gene gun inoculation was superior to vaccine delivery via needle and syringe. Based on these results, a DNA vaccine targeting the HTNV M segment was developed. Hamsters vaccinated with the HTNV DNA vaccine conferred sterile protection against HTNV, SEOV, and DOBV infection while nonhuman primates vaccinated with the HTNV DNA vaccine developed a robust neutralizing antibody response [54]. These successes led to the development of M segment DNA vaccines for PUUV [55], ANDV [56], and SNV [57].

A Phase 1 clinical trial was conducted to evaluate the safety and immunogenicity of HTNV and PUUV DNA vaccine when administered alone or in combination to healthy volunteers using gene gun [58]. There were no study-related adverse events; however, the groups vaccinated with HTNV or PUUV DNA vaccine alone developed neutralizing antibodies in 30% or 44% of group participants. Of participants that received the combined vaccine, 56% developed neutralizing antibodies to one or both viruses. While the vaccines were considered safe, an additional Phase 1 clinical trial (S-11-12 1854, NTCT01502345) was conducted with these vaccines in an effort to bolster immunogenicity. Vaccination of healthy volunteers with either HTNV, PUUV, or combined HTNV/PUUV DNA vaccines by intramuscular electroporation resulted in neutralizing antibodies detected in 56%, 78%, and 78% of participants, respectively [59]. These results are promising in that increased immunogenicity can be achieved by advances in vaccine delivery technology.

As of the writing of this manuscript, a Phase 2a clinical trial (S-14-01 2085, NTCT02116205) of 120 healthy volunteers to optimize the dose of HTNV, PUUV, and HTNV/PUUV DNA vaccines delivered by intramuscular electroporation is nearing completion, the final scientific report is in draft. A Phase 1 clinical trial (S-15-40 2289) of 27 healthy volunteers to evaluate the safety and immunogenicity of HTNV, PUUV, and HTNV/PUUV DNA vaccines delivered by the PharmaJet Stratis^®^ is also nearing completion, the final scientific report is in draft. Another Phase 1 clinical trial (16–0119, NTCT03682107) of 48 healthy volunteers to evaluate the safety and immunogenicity of the ANDV DNA vaccine is ongoing with the first cohort starting the vaccination series. Finally, a Phase 1 clinical trial (S-15-39, NCT03718130) of 72 healthy volunteers to evaluate the safety and immunogenicity of HTNV, PUUV, and HTNV/PUUV DNA vaccines delivered by intradermal and intramuscular delivery using the TriGrid Delivery System has been approved by an Institutional Review Board, but has not yet started recruiting. The evolution of hantavirus vaccine efforts is depicted in Table 1.

## 3. Hantavirus Disease Treatment Development

Herein, hantavirus disease treatments will be described based on the timing of drug administration in the hantavirus disease course. Drugs can be administered as either a postexposure prophylactic or a therapeutic. A postexposure prophylactic is administered after virus infection prior to the onset of viremia or clinical signs. A therapeutic is typically associated with a trigger-to-treat, either onset of clinical signs or detection of viremia. Candidate drugs evaluated in hantavirus animal models is described in Table 2.

### 3.1. Ribavirin

Initially found to inhibit HTNV replication in vitro [60], ribavirin administered to suckling mice after the onset of clinical signs reduced viral load and lethality by 45% compared to control animals [61]. Ribavirin treatment was optimized using the ANDV/hamster lethal HPS model. In one study, 100 mg/kg and 50 mg/kg of ribavirin protected hamsters from lethal HPS without toxicity [62]. It was also determined that ribavirin treatment starting up to 14 days post-ANDV intranasal challenge resulted in significant protection. In a second study, the postexposure efficacy of ribavirin treatment was confirmed with protection from lethal HPS disease observed when ribavirin treatment began 3 days following an intraperitoneal ANDV challenge [63]. Additionally, abbreviated treatment regimens (e.g., 3 days, 5 days, and 7 days) conferred protection when treatment commenced 1 day following virus challenge.

A clinical trial for the treatment of HFRS using ribavirin has been conducted. In this trial, ribavirin treatment was assessed in 242 patients with confirmed HFRS in China [64]. Morbidity and mortality were both significantly reduced confirming efficacy when administered postexposure (i.e., prior to the onset of clinical signs). In contrast, a clinical trial for the treatment of HFRS caused by PUUV infection conducted in Russia indicated that intravenous ribavirin did not alter viral load kinetics [65]. Two clinical trials for the treatment of HPS using ribavirin have been completed. Unfortunately, efficacy of intravenous ribavirin could not be assessed in either of these trials. Similar survival curves were observed in ribavirin-treated patients when compared to HPS patients during the same timeframe [66] or placebo-treated patients [67]. A similar conclusion reached in both of these trials is that once HPS progresses to the cardiopulmonary phase, ribavirin (used as a therapeutic) treatment is likely ineffective.

### 3.2. Lactoferrin

A single study evaluated the efficacy of the iron-binding glycoprotein, lactoferrin, to prevent HFRS in suckling mice was done [68]. While greatly limiting focus formation in vitro, a combination of lactoferrin and ribavirin was required to completely inhibit focus formation. In the in vivo SEOV/suckling mouse model, lactoferrin concentrations of 40 and 160 mg/kg administered as 2 doses resulted in 85% and 94% survival, respectively, when treatment began prior to virus challenge.

### 3.3. ETAR

ETAR (1-beta-d-ribofuranosyl-3-ethynyl-[1,2,4] triazole) is a nucleoside analog with antiviral activity against HTNV and ANDV in vitro [69]. In vivo efficacy was assessed in the HTNV/suckling mouse model. Ten days following HTNV challenge, mice were administered either 12.5 mg/kg or 25 mg/kg ETAR for 15 days resulting in a statistically significant increase in survival (25% and 26%, respectively, when compared to 10% survival in virus-only controls).

### 3.4. Favipiravir

Favipiravir is a pyrazine derivative initially discovered for its anti-influenza properties. This compound was first evaluated in vitro against a panel of bunyaviruses and arenaviruses, having antiviral activity against both viral families [70]. Favipiravir was evaluated in vivo and in both the SNV/hamster infection model and the ANDV/hamster lethal disease model. Oral twice daily administration of 100 mg/kg/day favipiravir reduced detection of SNV RNA in the blood and SNV antigen in the lungs [71]. Similarly, oral twice daily administration of 100 mg/kg/day favipiravir resulted in 100% survival, reduced detection of ANDV RNA in the blood and ANDV RNA and antigen in the lung. Consistent with other studies, delayed antiviral treatment after the onset of viremia was not protective in the ANDV/hamster model.

### 3.5. Vandetanib

Mechanisms of increased microvascular endothelial cell permeability due to hantavirus infection have been examined in vitro. ANDV enhances the permeability of endothelial cells in response to VEGF [72,73,74] and a 3D human lung tissue model showed increased expression of VEGF-A in response to ANDV infection [75]. As increased levels of VEGF have been noted in the plasma and serum of HPS patients [74,76], small molecules targeting vasoactive mediators are a novel way to repurpose FDA-approved drugs towards an alternative indication. Vandetanib is a tyrosine-kinase inhibitor targeting VEGF receptor 2 (VEGFR2) with the ability to prevent phosphorylation of VEGFR2 in vitro resulting in decreased VE-cadherin degradation [77]. In the ANDV/hamster model, hamsters treated with 10, 25, and 50 mg/kg/day starting 5 days before ANDV challenge delayed lethality and increased total survival by 23%. Similar small molecule drugs have been evaluated as a therapeutic (treatment starting after the onset of viremia) in the ANDV/hamster model with no effect [78].

### 3.6. Corticosteroids

Hantaviruses preferentially infect endothelial cells and induce host responses to include a proinflammatory cytokine response. While not an antiviral, it was thought that limiting this aspect of the immune response to virus infection may confer clinical benefit. The first evaluation of this was done during the Korean War. Cortisone therapy was administered by either the intramuscular or oral route [79]. There was no decrease in mortality, but it was determined that there was decreased lethality due to shock.

In Chile, a retrospective analysis of 22 HCPS patients treated with a high dose of methylprednisolone suggested that treatment reduced shock and mortality [80]. This led to the implementation of this treatment in some Chilean medical centers [81]. Subsequently, a phase 2 clinical trial to evaluate the safety and efficacy of intravenous methylprednisolone for the treatment of HPS in the cardiopulmonary phase was conducted. The results of this trial indicate that methylprednisolone does not confer clinical benefit [81].

### 3.7. Immunotherapy, Monoclonal Antibodies

In the 1980s, several monoclonal antibodies to HTNV were characterized describing neutralization sites on the glycoproteins, G_n_ and G_c_ [82,83,84]. In an effort to correlate specific viral epitopes with protection from HTNV infection, monoclonal antibodies were evaluated by passive transfer [35]. This pivotal experiment demonstrated that of 15 monoclonal antibodies tested, a neutralizing antibody response to either G_n_ or G_c_ alone is sufficient to prevent HTNV infection in hamsters. Protective efficacy of the monoclonal antibody HCO2 was confirmed in a subsequent HTNV/hamster experiment [85]. The finding that neutralizing monoclonal antibodies were protective was also confirmed later in a suckling mouse/HTNV model [86].

Recently, researchers have shown that two recombinant monoclonal antibodies generated from an ANDV convalescent survivor with high neutralizing antibody titers protected ANDV-infected hamsters from lethal disease [87]. In this study, convalescent sera from 27 ANDV survivors was screened and a single patient with high neutralizing antibody titer was selected. Recombinant antibodies were produced from ANDV glycoprotein-specific memory B cells and two monoclonal antibodies, JL16 and MIB22, effectively neutralized ANDV in vitro. In vivo, passively transferred antibodies on days 3 and 8 postinfection prevented lethality in ANDV intranasally infected hamsters when administered either alone or in combination.

### 3.8. Immunotherapy, Polyclonal Antibodies

Human convalescent plasma has been shown to protect animals in both the SNV/deer mouse infection model [88] and the ANDV/hamster lethal disease model [89] demonstrating that neutralizing antibody is sufficient to protect from infection and disease. When translating these finding to the clinic, in a nonrandomized multicenter trial of human immune plasma for treatment of HPS caused by ANDV, researchers in Chile discovered intravenous infusion of 5000 NAU/kg resulted in borderline statistically significant clinical benefit [90]. The authors hypothesize that earlier treatment, as demonstrated in the ANDV/hamster model, would likely increase positive outcomes. The need for ABO blood typing of convalescent plasma and the lack of a standardized product are two main limiting factors to this therapy.

To this end, the development of a standardized polyclonal antibody product using hantavirus DNA vaccines is currently in-progress. Hantavirus DNA vaccines have been shown to be immunogenic in rabbits, NHPs, ducks, and geese [53,54,55,56,89,91,92,93]. Most recently, a fully human polyclonal antibody product generated in ANDV DNA and SNV DNA vaccinated transchromosomal bovine protects in two lethal HPS animal models [94]. Current work is underway to expand this research to create a standardized polyclonal antibody product against ANDV, SNV, HTNV, and PUUV that can move forward through preclinical testing towards a Phase 1 clinical trial.

## 4. Conclusions

The number of hantavirus disease cases each year warrants the development of medical countermeasures to combat infection from these viruses. However, the number of cases each year do not necessitate a widespread vaccination strategy outside of endemic areas except in the case of military conducting operations in these areas. Therefore, it is imperative to work towards the goal of a vaccine and an antiviral that could be used individually or in combination. An antiviral, such as a polyclonal antibody therapy, would provide instant immunity while vaccination could provide long-lasting immunity. Vaccination would also prevent the spread of ANDV, the only hantavirus with demonstrated person-to-person transmission. Antiviral treatment and vaccination is a strategy currently used for rabies virus infection that could be applied to hantavirus infections.

The timing of treatment intervention presents a challenge for hantavirus disease, especially cases of HPS. Animal models have repeatedly shown that antivirals are not effective if administered after the onset of viremia. The prodrome phase can be difficult to differentiate from other febrile illnesses, hampering early intervention efforts. Continued work is required to determine if there are early host responses that can be used to support a hantavirus diagnosis.

Additionally, the lack of an HFRS disease model that faithfully recapitulates the salient features of human HFRS disease is hampering medical countermeasure development efforts. Current infection models have utility for testing vaccines and antivirals in which sterile immunity can be used as an output. The unavailability of a model that can be used to evaluate antivirals targeting specific aspects of pathogenesis, postexposure prophylactics, or therapeutics is a major shortcoming of the field.

The advancement of several vaccines into clinical trials is promising for the future use of these vaccines as licensed products or in emergency use. There are several path-to-licensure issues that must be overcome, reviewed in [95]. These include limited site for Phase III efficacy trials, lack of significant government and industry investments, and current limitations to the use of “animal rule.” Continued research efforts are necessary to propel more antiviral products towards clinical trials. The need for these medical countermeasures perpetuates as outbreaks around the world continue to occur.

## Figures and Tables

**Table 1:** Hantavirus candidate vaccines. ANDV: Andes virus; DOBV: Dobrava virus; HTNV: Hantaan virus; PUUV: Puumala virus; VACV: vaccinia virus; VSV: Vesicular stomatitis virus.

Candidate Vaccine	Vaccine Type	Date of Last Publication
VACV-vectored HTNV (USA)	Virus-vectored recombinant	2000
PUUV N, ANDV N, DOBV N (Sweden)	Recombinant N protein	2002
DOBV N (Germany)	Recombinant N protein	2004
HBcdDOB120, HBcdHTN120, HBcdPUU120 (Germany)	Chimeric core particles	2005
Nonreplicating VSV/HTNV pseudotype (Japan)	VSV pseudotype	2006
DOBV N, PUUV N (Belgium)	Recombinant N protein	2008
Ad-vectored ANDV (USA)	Virus-vectored recombinant	2009
VSV-vectored ANDV (USA)	Virus-vectored recombinant	2011
HTNV DNA, PUUV DNA (gene gun, USA)	Nucleic acid-based molecular	2012
pVAX-LAMP/Gn, pVAX-LAMP/Gc (China)	Nucleic acid-based molecular	2018
HFRS Vaccine (China)	Inactivated	2018
Hantavax^®^ (Republic of Korea)	Inactivated	2019
CD40L and GM-CSF HTNV VLPs (China)	VLP	2019
HTNV DNA, PUUV DNA (IM-EP, USA))	Nucleic acid-based molecular	2014 and ongoing clinical trial (S-14-01 2085, NTCT02116205)
HTNV DNA, PUUV DNA (PharmaJet Stratis^®^, USA)	Nucleic acid-based molecular	Ongoing clinical trial (S-15-40 2289)
ANDV DNA (PharmaJet Stratis^®^, USA)	Nucleic acid-based molecular	Ongoing clinical trial (16–0119, NTCT03682107)
HTNV DNA, PUUV DNA (IM-EP, ID-EP, USA)	Nucleic acid-based molecular	Ongoing clinical trial (S-15-39, NCT03718130)

**Table 2:** Hantavirus candidate drugs.

Candidate Drug	Drug Type	Date of Last Publication
Ribavirin	Nucleoside analog	2017
Lactoferrin	Iron-binding protein	2001
ETAR	Nucleoside analog	2008
Favipiravir	Pyrazine derivative	2013
Vandetanib	Tyrosine kinase inhibitor	2016
Methylprednisone	Corticosteroids	2013
JL16, MIB22	Monoclonal antibodies	2018
Human convalescent plasma	Polyclonal antibodies	2015
Purified human plasma from transchromosomal bovine	Polyclonal antibodies	2014
